# New Immunosensing-Fluorescence Detection of Tumor Marker Cytokeratin-19 Fragment (CYFRA 21-1) Via Carbon Quantum Dots/Zinc Oxide Nanocomposite

**DOI:** 10.1186/s11671-020-3247-9

**Published:** 2020-01-15

**Authors:** Nawal Ahmed Alarfaj, Maha Farouk El-Tohamy, Hesham Farouk Oraby

**Affiliations:** 1 Department of Chemistry, College of Science, King Saud University, P.O. Box 22452, Riyadh, 11495 Saudi Arabia; 2 Deanship of Scientific Research, Umm Al-Qura University, Makkah, Saudi Arabia

**Keywords:** Carbon quantum dots, Cytokeratin fragment 21-1 antigen, Zinc oxide nanoparticles, Immunoassay, Fluorescence

## Abstract

The rapid detection of lung cancer in early stages using the antigen cytokeratin-19 fragment (CYFRA 21-1) as a tumor marker in human serum plays an important role in the survival of patients and taking a fast surgical reaction. This study aimed to employ the green synthesized carbon quantum dots conjugated zinc oxide nanocomposite as a highly sensitive fluorescence immunosensing solution for fast determination of CYFRA 21-1 antigen in human serum. The suggested method was conducted by applying a hydrothermal method to prepare carbon quantum dots using *Citrus lemon* pericarp. The formed carbon quantum dots were used in the reduction and stabilization of zinc acetate to synthesize carbon quantum dots-zinc oxide nanocomposite. To form a sandwich capping antibody-antigen-antibody immunosensing system, a CYFRA 21-1 antigen was trapped by immobilizing a non-conjugated monoclonal antibody BM 19.21 on the surface of carbon quantum dots-zinc oxide nanocomposite and another monoclonal antibody KS 19.1, which was coated on the microtiter well surface. This system has a tunable fluorescence feature recorded at excitation and emission of λ_ex_ = 470 and λ_em_ = 520 nm, respectively. The suggested nanocomposite fluorescence immunosensing system displayed a linear relationship of 0.01–100 ng mL^−1^ with a limit of detection of 0.008 ng mL^−1^. The suggested immunosensing system based on carbon quantum dots-zinc oxide nanocomposite provides a promising approach for rapid diagnoses of lung cancer by detecting CYFRA 21-1 in human serum.

## Introduction

Lung cancer is the most public and aggressive cancer type with great challenges in medical treatment. Tumor recurrence and metastasis are considered the major leading causes of death for lung cancer patients [1]. The tumor marker cytokeratin 19 fragment (CYFRA 21-1) is a fragment that exists in many normal as well as malignant epithelial cells [2]. It can be estimated using a sandwich immunoradiometric assay. The early studies clarified that in malignant stages of lung cancer, CYFRA 21-1 is released into the blood circulation of patients and elevated in their serum [3]. Therefore, it is possible to improve the survival of lung cancer patients by early detection and consequently fast surgical reaction [4].

Few techniques were previously reported for the detection of CYFRA 21-1, including enzyme immunoassay [5], electrochemiluminescence immunoassay [6], and immunoradiometric assay [7]*.* An advantageous strategy for enhancing and improving the sensitivity of CYFRA 21-1 in human serum is still a concern.

In recent years, major progress and explosive growth of nanotechnology has been achieved in almost all life fields [8]. Among those fields are drug delivery systems [9], pharmaceutical analysis [10], catalytic activity reactions [11], medicinal applications [12], cancer tumor markers [13], and tissue imaging [14].

Nowadays, fluorescence (FL)-based sensing techniques have attracted many researchers due to their simple design and excellent sensitivity. Various FL sensory materials have been designed and synthesized for biological monitoring. The FL systems for biological determination are highly luminescent, water-dispersible, chemically stable, and nontoxic [15]. There are various immunosensing fluorescence-based probes for biomarker detection. The heterogeneous competitive assay is conducted by immobilizing capture molecules on the surface and then incubated with fluorophore-conjugated biomarkers. The competition between the free and conjugated biomarkers for binding to the capture molecules decreases the fluorescence intensity with biomarker concentration [16]. The heterogeneous sandwich assay is based on the incubation of capture molecules and solution of interest forming a complex with biomarkers. Consequently, the fluorescence intensity increases with biomarker concentration [17].

In the homogeneous competitive assay, two different fluorophore A-conjugated capture molecules conjugated with fluorophore B-conjugated biomarkers and the solution increasing the fluorescence with biomarker concentrations [18]. However, those techniques showed certain drawbacks, including their long experimental time, lack of multiplexed detection, complexity, and sometimes relatively false results. Advancement in nanotechnology enabled researchers to develop novel fluorescence immunosensing probes with unique optical characteristics [19]. Since the first use of quantum dots in biomolecule detection, they have gained a great deal of interest as their optical features provide high flexibility in the selection of suitable wavelength, excellent labels for multiplexed detection, biocompatibility, and targeting capacity [20].

Carbon quantum dots (CQDs) have demonstrated excellent chemical, physical, optical, magnetic, and electrical properties. CQDs can be synthesized using different techniques, including hydrothermal, electro-oxidation, laser ablation, and microwave methods [21–24]. Due to their low toxicity features, scientific researchers considered CQDs as powerful candidates in many fluorescent probes. Additionally, they have a strong ability to manipulate through different controllable chemical reactions in various demands such as biochemical, photochemical, biosensing, bioimaging, and drug delivery systems [25–27], as well as in immunoassay detection [28]. Earlier studies on the synthesis of CQDs revealed certain disadvantages by using expensive carbon sources, toxic chemicals and reagents, or using non-selective processes [29]. To restrict those disadvantages, researchers started using fruit juices as novel and cheap source of carbon [30]. Since the use of fruit juices does not provide the optimal goal of utilizing resources, fluorescent CQDs were recently obtained from fruit peels [31]. The use of fruit peels provides a promising route for eco-friendly and green synthesis of CQDs.

Zinc oxide (ZnO) is one of the most important, potentially active, stable and low toxic metal oxides that widely used in ultraviolet laser devices, biomedical field, various types of sensors, and photocatalysis [32–35]. ZnO nanoparticles (ZnONPs) displayed photoluminescent properties near UV and Vis spectrum ranges. This can be attributed to the excitonic emission which is based on the direct recombination of electron-hole pairs [36] or due to the green-yellow emission at 520 nm as a result of the electronic transition from the conduction band edge to a trap level [37].

Generally, carbon dots are amorphous or nanocrytalline quasi-spherical nanoparticles containing sp^2^ and sp^3^ carbon, O/N-based groups, and post modified chemical groups. Furthermore, CQDs have the ability to excite with higher wavelengths and can change the efficacy of the combined surfaces of the electron-hole pairs and treats the quenching in the analyzed systems, which may facile the quantitative determination of biomolecules [38]. They have the ability to be decorated by metal oxides such as TiO_2_ and ZnO to form optically active nanocomposite that can be exploited in the detection of biomarkers in human serum. ZnO is a material of wide band gap (3.37 eV), which can be luminescent in UV and blue regions of the visible light due to the presence of a large density of defect levels in the band gap [39]. The formation of CQDs/ZnO nanocomposite increases the visible light absorption due to the hybridization of ZnO with CQDs, and the blue shift in the luminescence absorption to 520 nm can be attributed to the radiative recombination of ionized O vacancies. In addition to the increase in absorption of visible light, a better electron-hole separation and reduction of interfacial electron transfer time may be considered for the higher optical performance of the hybridized CQDs with ZnO nanoparticles [40]. Furthermore, the meaningful increase of –OH* radicals, generated from CQDs/ZnO nanocomposite in the water interface, can cause a significant elevation in fluorescence signals of the analyzing system. Thus, the combined CQDs/ZnO nanocomposite enhances the modification of optoelectronic and photoluminescence properties of ZnO surface and produces a strong surface defect with tunable photoluminescence [41]. Moreover, CQDs immobilized with bio-recognition antibodies forming antibody-antigen-antibody FL-sensing system provide a viable probe with high specificity and sensitivity to the target analyte [42].

The suggested study proposed a new simple and ultrasensitive immunoassay fluorescence sensing system based on CQDs decorated with ZnO nanocomposite to determine the tumor CYFRA 21-1 marker in human serum. Citrus lemon pericarp was employed as a carbon precursor to derive CQDs using hydrothermal conditions. Moreover, it was used as a reducing and stabilizing agent for the synthesis of CQDs conjugated ZnO nanocomposite. The prepared CQDs/ZnO nanocomposite was immobilized by a non-conjugated BM 19.21 monoclonal antibody (mAb) and the microtiter wells were coated with another monoclonal KS 19.1 antibody to form a sandwich capping immunosensing system.

## Methods

### Instruments

Spectrophotometric spectra of both CQDs as well as CQDs/ZnO nanocomposite were recorded using an Ultrospec 2100-Biochrom spectrophotometer (Biochrom Ltd., Cambium, Cambridge, UK). Surface morphology and particle size distribution of the green synthesized CQDs and CQDS/Zn nanocomposite were evaluated using a transmission electron microscope (TEM) JEOL 1200EX model instrument (JEOL Ltd., Freising, Germany) and scanning electron microscope (SEM) JSM-7610F model (JEOL, USA). The fluorescence and Fourier transform infrared (FT-IR) spectra of the suggested immunosensing system were checked using Biotek Synergy H1 multi-mode reader (Biotek, Tokyo, Japan) and Perkin Elmer FT-IR spectrophotometer (PerkinElmer Ltd., Yokohama, Japan), respectively. Raman spectra, X-ray photoelectron spectroscopy (XPS) and X-ray powder diffraction (XRD) pattern were measured using micro-Raman spectrometer (CRAIC Technologies, CA, USA), Kratos Axis Ultra X-ray spectroscopy system (Kratos Analytical Ltd., Manchester, UK) and Siemens D-5000 diffractometer (Siemens, Erfurt, Germany), respectively.

### Chemicals and Reagents

SG-2000-10090 instrument (Barsbuttel, Germany) was used to acquire the deionized water used throughout all experiments. CYFRA 21-1-non-conjugated monoclonal antibodies (mAb) BM 19.21 and KS 19.1 to form the sandwich capping immunosensing system were obtained from Abcam (Cambridge, UK). *Citrus lemon* fruits were supplied by local markets. Phosphate-buffered saline (PBS) of pH = 7.4 was prepared using sodium chloride, potassium chloride, sodium hydroxide, monopotassium phosphate, and disodium phosphate (BHD Ltd. Co. Poole, UK). Randox Laboratories (Northern Ireland-UK) kindly provided the commercial normal sera. Random blood samples were collected from healthy volunteers, and prior to start this study, an informed consent was obtained. Furthermore, Sigma-Aldrich (Hamburg, Germany) supplied a pure grade of both carbodiimide hydrochloride (EDC) and N-hydroxysuccinimide (NHS). The research ethics committee at King Saud University, KSA (KSU-REC-002-E, 2019) approved the study.

### Green Hydrothermal Preparation of Carbon Quantum Dots (CQDs)

**Scheme 1:**
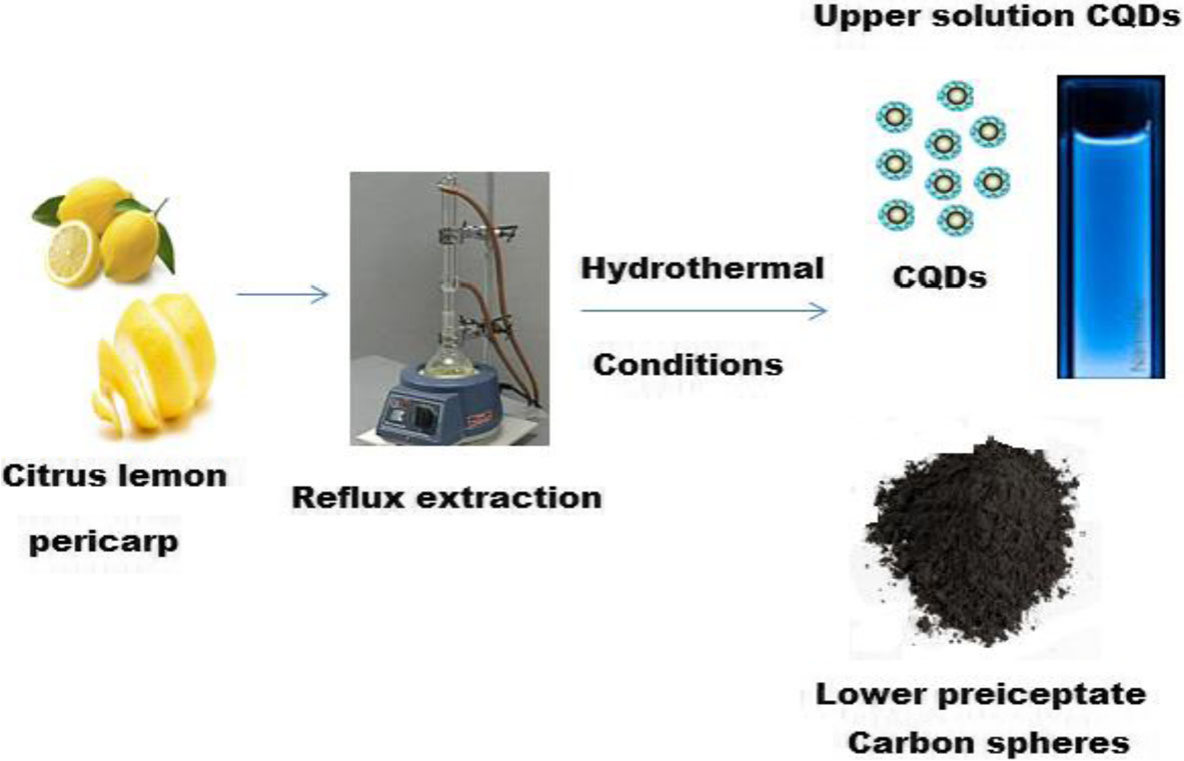
Green synthesis of CQDs using *Citrus lemon* pericarp to fluorescent CQD solution and carbon spheres

*Citrus lemon* pericarp was employed to synthesize CQDs under hydrothermal conditions. Approximately 20 g of *Citrus lemon* pericarp and 200 mL of deionized water were transferred to a rounded flask and refluxed at 100 °C under continuous magnetic stirring for 6 h. After cooling to room temperature, the resulted extract was centrifuged at 3500 rpm and 20 mL of the upper extracted solution was autoclaved and heated under hydrothermal conditions in the temperature range from 100 to 200 °C for different intervals of 6–120 h. After cooling to room temperature the upper liquor represents the CQDs (Scheme 1).

### Preparation of Carbon Quantum Dots-Zinc Oxide Nanocomposite

To prepare CQDs/ZnO nanocomposite, a simple chemical reduction reaction was performed using CQDs as a reducing and stabilizing agent. CQDs/ZnO nanocomposite was obtained by adding 20 mL of the CQDs to 50 mL of 5.0 × 10^−2^ mol L^−1^ of zinc acetate at 60 °C under continuous stirring for 10 min. When the color of the mixture changed from yellowish to creamy, the mixture was left aside for 30 min to complete the reduction process and stored at 4 °C. To ensure stability and check the agglomeration of the prepared CQDs/ZnO nanocomposite, UV-Vis spectrometry was employed to record the absorbance within 20 days at 390 nm. The outcome results revealed high stability and no significant change in the absorbance of CQDs/ZnO nanocomposite.

### Characterization of Carbon Quantum Dots-Zinc Oxide Nanocomposite

To ensure the formation of CQDs/ZnO nanocomposite, different microscopic and spectroscopic techniques were used. The uniformity and surface morphology of CQDs and CQDs/ZnO nanocomposite were studied using high-resolution transmission electron microscope (HRTEM) and SEM. The optical spectra were studied using UV-Vis, FT-IR, XPS, and Raman spectroscopy. The crystal structure of the as-prepared CQDs was evaluated using XRD pattern.

### Immobilization Process

**Scheme 2:**
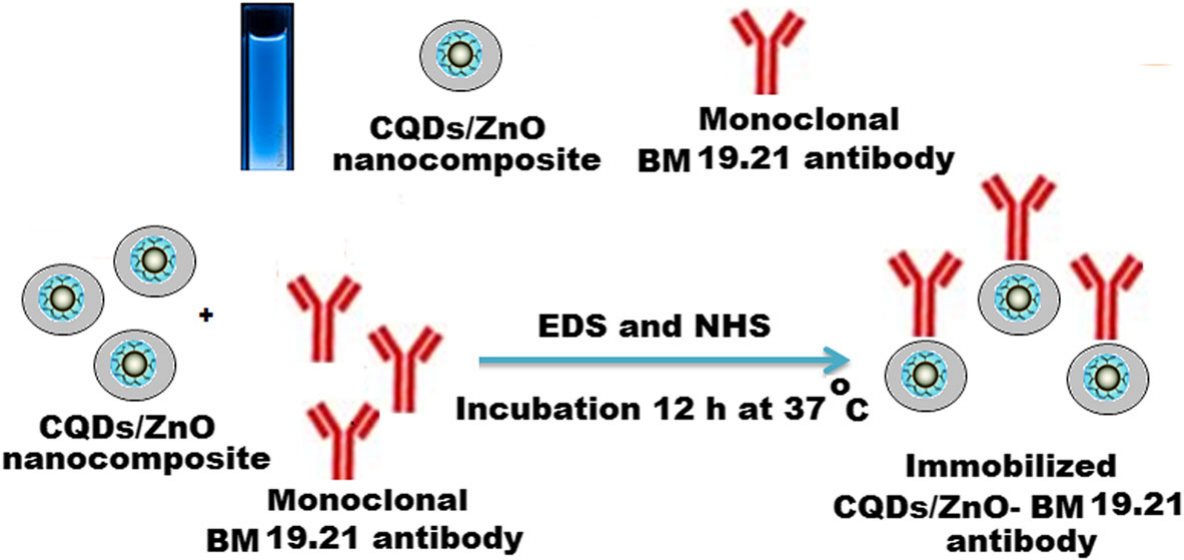
Immobilization of monoclonal BM 19.21 antibody on the surface of CQDs/ZnO nanocomposite

A non-conjugated monoclonal BM 19.21 antibody was immobilized on the surface of the synthesized CQDs/ZnO nanocomposite by a simple peptide amide bond between the amine and the active carboxylic groups. The immobilization process was conducted by adding 5.0 mL of each equimolar 3.0 × 10^−3^ mol L^−1^ NHS and EDC to 5.0 mL of CQDs/ZnO nanocomposite solution under continuous stirring for 1 h. Approximately 5 mg of non-conjugated monoclonal BM 19.21 antibody was dissolved in 1.0 mL of 0.01 mol L^−1^ phosphate-buffered saline (pH = 7.4) and added to the above-sensing solution. The non-conjugated monoclonal BM 19.21 antibody was immobilized on the surface of a CQDs/ZnO nanocomposite solution after incubation at 37 °C for 12 h (Scheme 2). Spectrophotometry was used to confirm the success of the immobilization process.

### General Principle of the Immunoassay Method

**Scheme 3:**
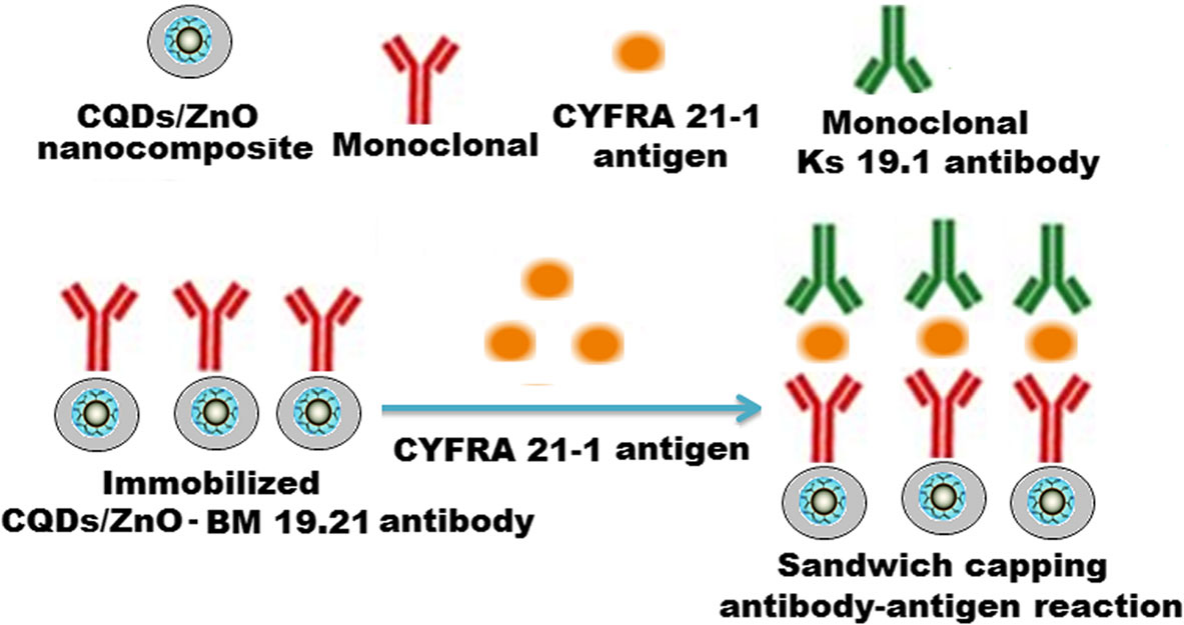
Illustrated scheme represents a sandwich capping immunosensing antibody-antigen-antibody reaction

A sandwich capping reaction antibody-antigen-antibody was obtained using another monoclonal KS 19.1 antibody coating the surface of microtiter wells (Scheme 3). Under optimal immunoassay conditions, the concentration of CYFRA 21-1 antigen was determined as a function of an increase in fluorescence signal intensity.

### The Immunosensing Procedure

The specimen collection of human sera was provided by random volunteers. Complete clotting was ensured before centrifugation at room temperature and stored at 4 °C. The spiking technique was used to prepare standard samples containing CYFRA 21-1 antigen in the concentration range of 0.01–500 ng mL^−1^. Approximately 50 μL of the spiked samples were dispensed in microtiter wells and mixed with 50 μL freshly diluted monoclonal KS 19.1 antibody using phosphate-buffered saline of pH = 7.4 for 30 min and then incubated without covering the plate for 1 h at 37 °C. The content of the wells was shaken out briskly, and the wells were rinsed three times using deionized water (300 μL) for each well. Approximately 50 μL of the immobilized CQDs/ZnO-BM 19.21 nanocomposite solution was added to each well, gently mixed, and incubated for 30 min at 37 °C. The prepared samples were subjected to fluorescence analysis using microtiter reader to record the intensities.

## Results and Discussion

### Morphological Evaluation of Carbon Quantum Dots and its Nanocomposite

**Fig. 1:**
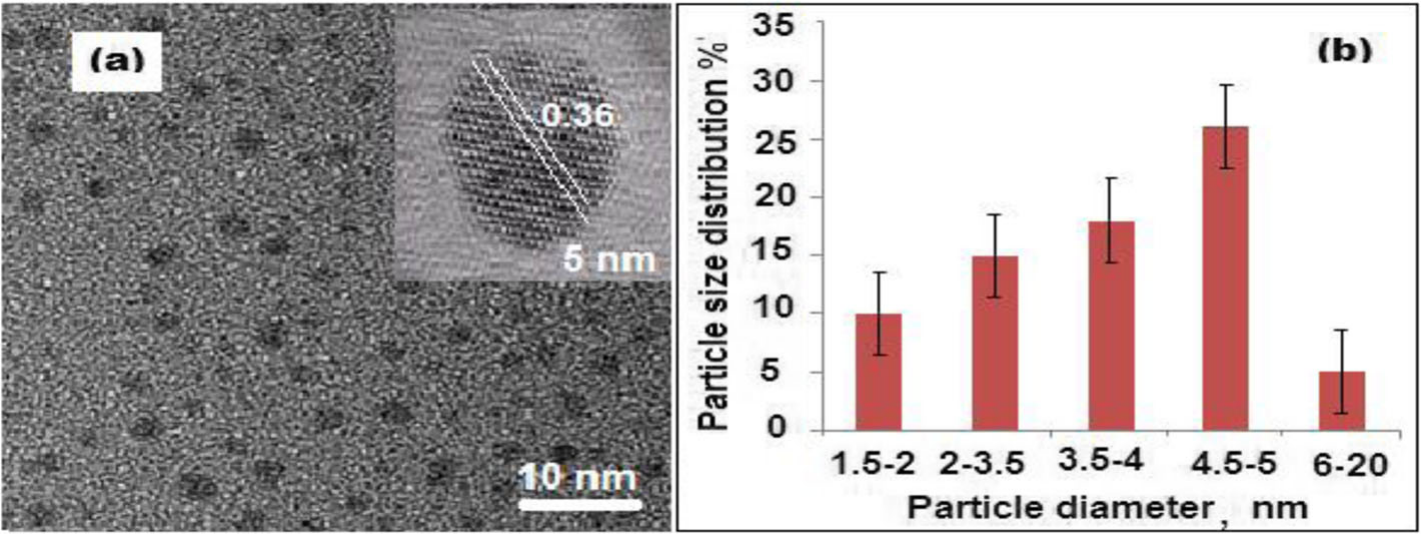
**a** High-resolution transmission electron microscope (HRTEM) image of CQDs with diameter 5 nm and **b** size distribution graph of the CQDs based on the TEM

A transmission electron microscope (TEM) was used to characterize the surface morphology and the distribution of CQDs in the samples. To accomplish the examination under TEM, approximately 4 μL of the prepared CQDs suspension was dropped on the surface of the carbon grid of TEM. In HRTEM image (Fig. 1a), the observed uniform black spots with lattice spacing (0.36 nm) indicated the formation of CQDs. A particle size distribution graph was plotted and the average particle size ranged from 1.5 ± 0.5 to 5.0 ± 0.5 nm (Fig. 1b). The obtained particle size proved that the formed CQDs are indeed the quantum-sized nanomaterials. Moreover, a dynamic light scattering (DLS) was carried out, and the average particle size was found to be ~ 20 ± 0.2 nm. A difference between the previous two measurements was observed. Previous studies revealed that HRTEM does not show the crystal lattice structure of the formed CQDs at higher magnifications due to their amorphous nature [43]. Similarly, in this study, the natural precursor of carbon is *Citrus lemon* pericarp and the derived CQDs also exhibited amorphous nature. Therefore, the difference in particle size measurements can be attributed to the agglomeration of the formed CQDs, the amorphous nature of formed carbon dots, the mechanism involved in each experiment, and the hydration dynamics of the particles.

**Fig. 2:**
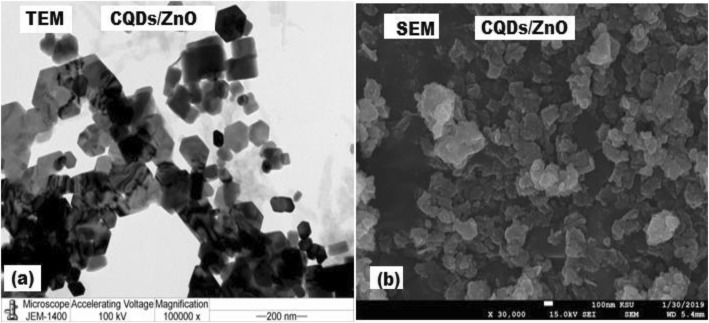
**a** and **b** represent the transmission electron microscope and scanning electron microscope images of CQDs/ZnO nanocomposite

The prepared CQDs/ZnO nanocomposite was investigated using TEM and SEM. In the TEM image (Fig. 2a), the presence of hexagonal particles attached to CQDs indicated the formation of CQDs/ZnO nanocomposite. In SEM, the nanocomposite sample was coated with gold to prevent the electron absorption by the sample and buildup of charge. The applied accelerated voltage was 15 kV at magnification × 30,000 (Fig. 2b).

**Fig. 3:**
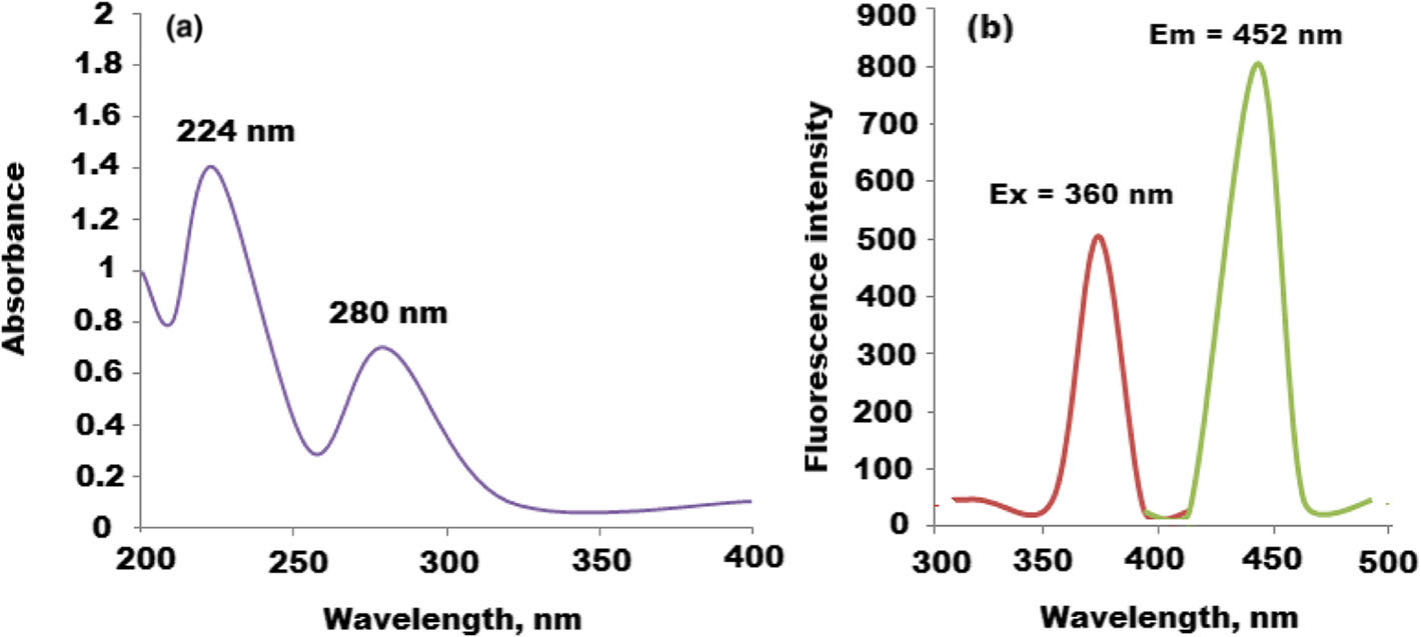
Spectroscopic spectra of CQDs (**a**) UV-Vis spectrum at 224 and 280 nm and (**b**) fluorescence spectrum of CQDs at λ_ex_ = 360 and λ_em_ = 452 nm

**Fig. 4:**
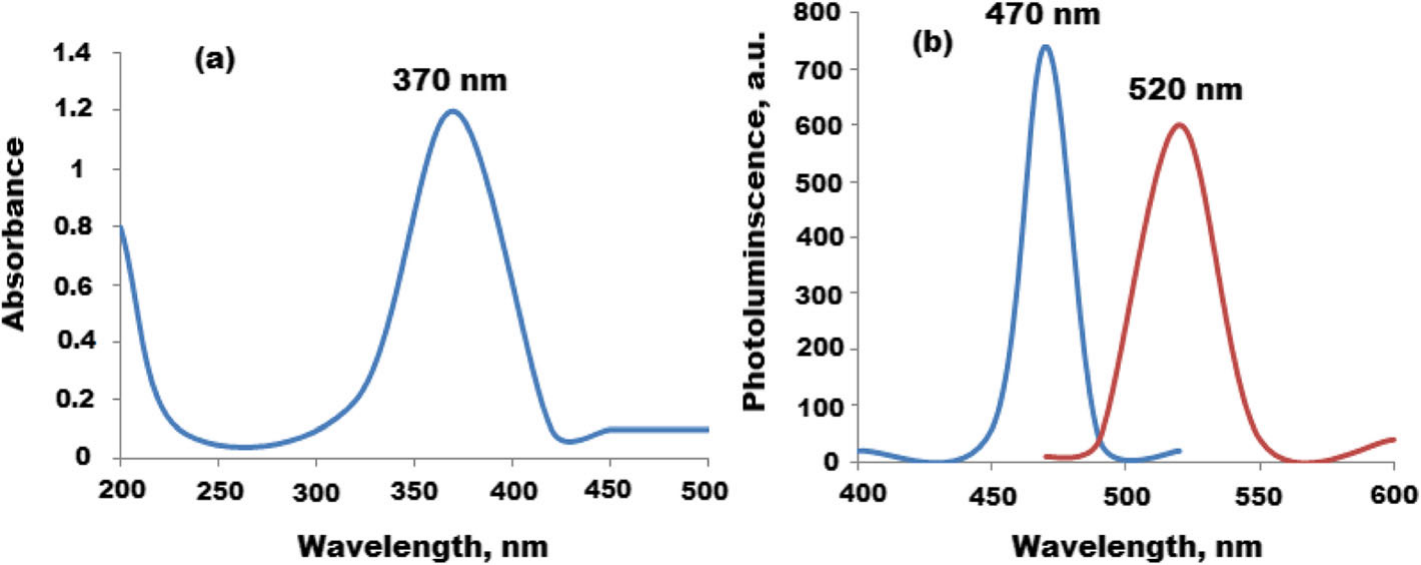
Spectroscopic spectra of CQDs/ZnONPs **a** UV-Vis spectrum at absorption peak at 370 nm and **b** photoluminescence spectrum of CQDs/ZnONPs at λ_ex_ = 470 and λ_em_ = 520 nm

UV-Vis and fluorescence spectra of CQDs were studied, and the recorded spectra showed two significant peaks at 224 and 280 nm which can be attributed to *p*~p* and *n*~P* transition of C=C and C=O, respectively. Also, the fluorescence spectrum of CQDs displayed two signals at maximum λ_ex_ = 360 and λ_em_ = 453 nm (Fig. 3a, b). Furthermore, the UV-Vis spectrum of CQDs/ZnO nanocomposite was studied. A significant absorption peak was observed at 370 nm displaying blue green shift (Fig. 4a). The photoluminescence (PL) properties of CQDs/ZnO nanocomposite were studied. The size and surface defects of CQDs greatly affect their luminescence properties. As a function of excitation wavelength, the (PL) emission of CQDs was varied [38]. Also, ZnO-nanosized particles exhibited a defect-related emission in the blue to green absorption visible region [41]. Therefore, CQD-decorated ZnONPs produced an excellent nanocomposite for PL emission. As shown in Fig. 4b, the PL spectrum of CQDs/ZnO exhibited a blue shift with a significant peak at 520 nm after excitation wavelength 470 nm. The observed shift can be attributed to the overlapping between the energy bands of CQDs and ZnONPs. The displayed blue shift was in the defect emission level 2.1 eV.

**Fig. 5:**
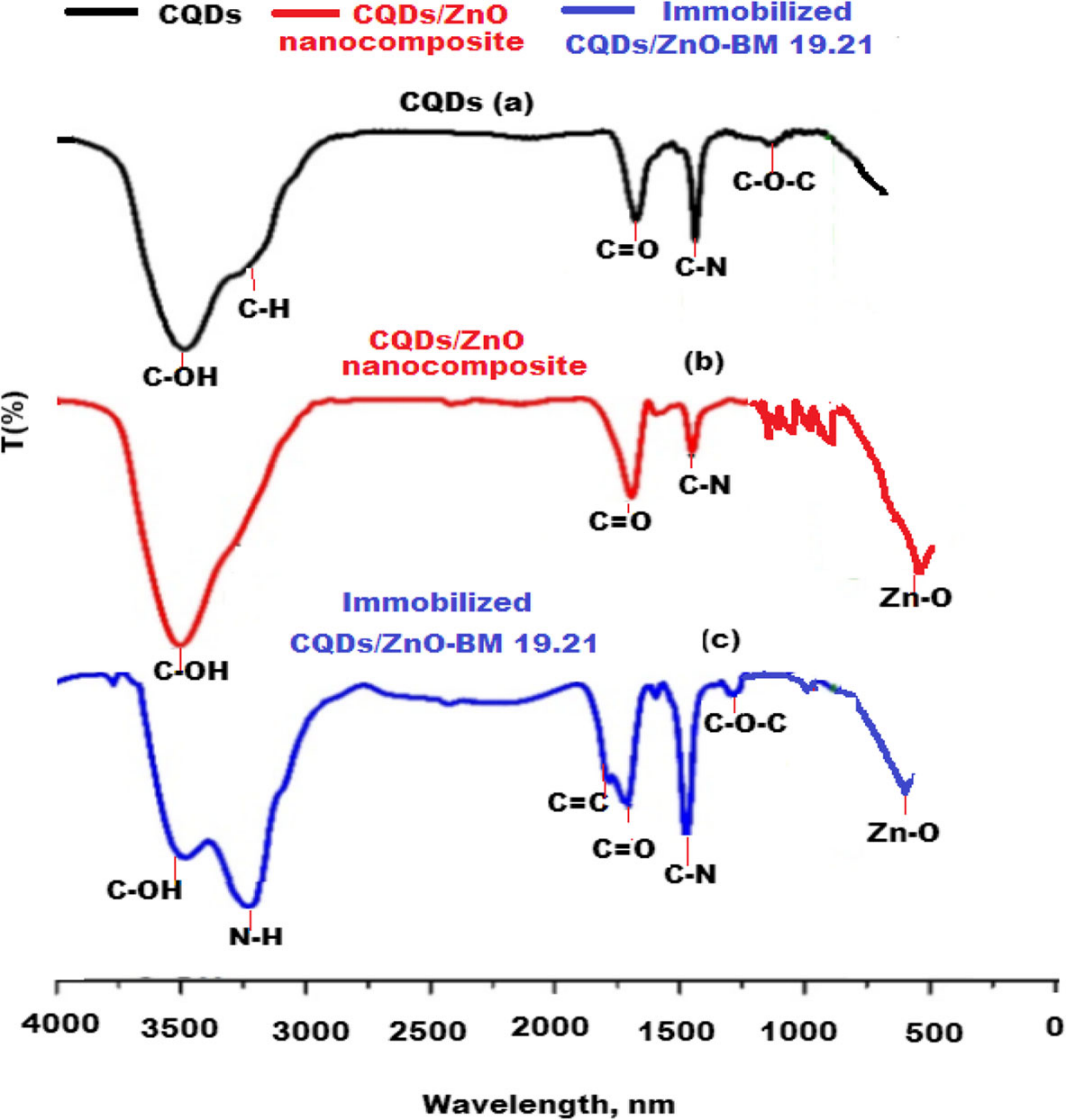
FT-IR spectra of **a** CQDs, **b** CQDs/ZnO nanocomposite, and **c** immobilized CQDs/ZnO-BM 19.21 nanocomposite

To confirm the formation of CQDs/ZnO nanocomposite and immobilized CQDs/ZnO nanocomposite with non-conjugated monoclonal BM 19.21 antibody, a comparative FT-IR study was performed. The recorded FT-IR spectrum of CQDs revealed the presence of different distinct peaks corresponding to certain functional groups, including stretching vibrational peaks at 3462 cm^−1^ and 2932 cm^−1^ for C–OH and C–H groups, respectively. Furthermore, three vibration absorption bands were observed at 1749 cm^−1^, 1375 cm^−1^, and 1246 cm^−1^ corresponding to the presence of C=O, C–N, and C–O–C functional groups, respectively (Fig. 5a). A new peak at 436 cm^−1^ corresponding to a stretching vibration band of Zn–O was observed. The reducing and stabilizing properties of CQDs were gained from the presence of –OH and COOH groups on their surface. These functional groups act as electron donors and have a strong affinity towards the formation of CQDs/ZnO nanocomposite. Therefore, CQDs reduced and stabilized the formed nanocomposite (Fig. 5b). As depicted in Fig. 5c, it was noticed that two new peaks were formed at 3254 cm^−1^ and 1675 cm^−1^. These peaks were attributed to stretching vibration of N–H and C=O, respectively, and confirming the immobilization of CQDs/ZnO-BM 19.21 via peptide bonds.

**Fig. 6:**
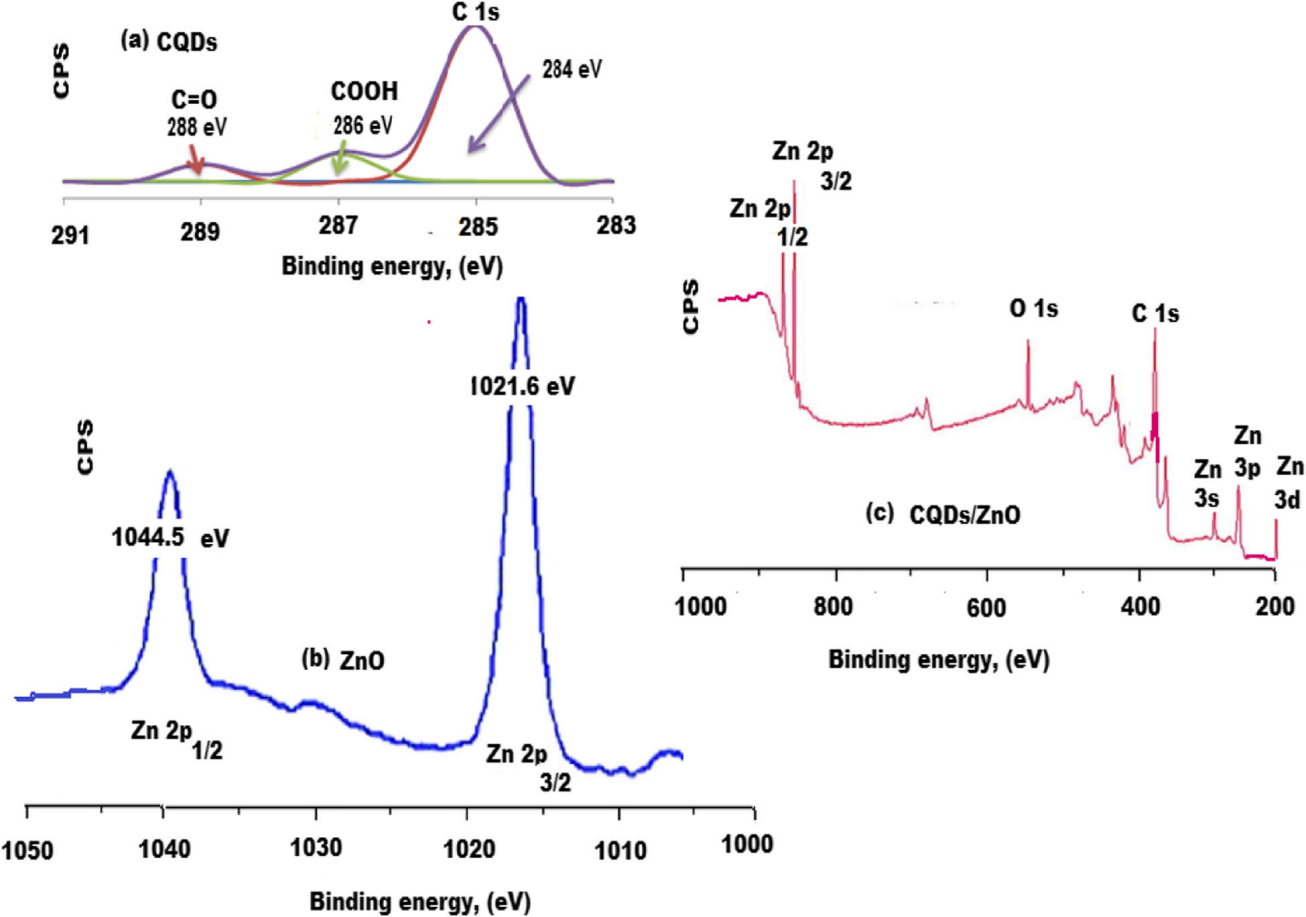
X-ray photoelectron spectroscopy (XPS) spectra of **a** CQDs, **b** ZnO, and **c** CQDs/ZnO nanocomposite

X-ray photoelectron spectroscopy (XPS) spectra of the green synthesized CQDs were examined. The obtained spectrum of CQDs (Fig. 6a) showed different functional groups at 288 and 286 eV for C=O and COOH, respectively. Also, two significant binding energy peaks were observed at 1044.4 and 1021.5 eV for Zn 2p_1/2_ and Zn 2p_3/2_, respectively (Fig. 6b). Moreover, the high-resolution XPS spectrum of CQDs/ZnO nanocomposite confirmed the presence of different binding energy peaks at 560, 385, 350, 246, and 200 eV for O 1s, C 1s, Zn 3s, Zn 3p, and Zn 3d, respectively (Fig. 6c). All previously mentioned data proved the presence of ZnO on the surface of CQDs forming CQDs/ZnO nanocomposite.

**Fig. 7:**
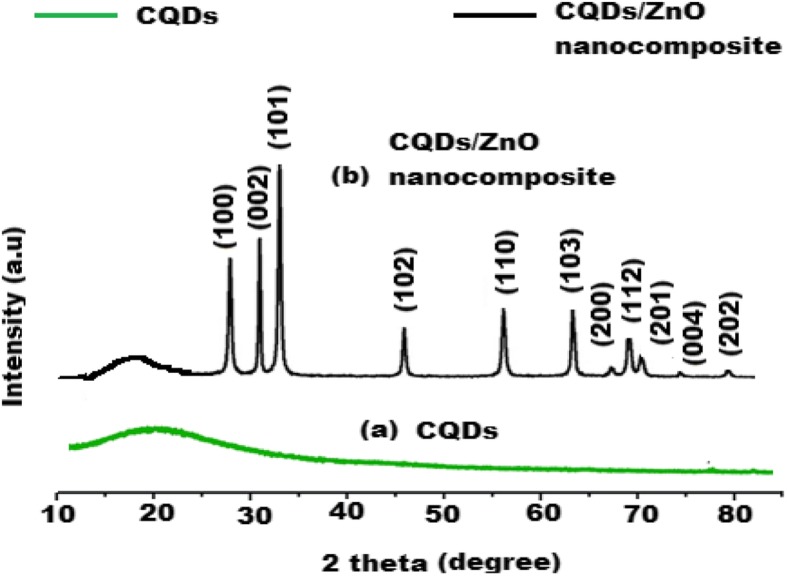
X-ray diffraction pattern of **a** CQDs and **b** CQDs/ZnO nanocomposite

A comparative study between XRD patterns of CQDs and CQDs/ZnO nanocomposite was performed. A broad peak at 20° (2Ɵ) for carbon dots was noticed in the XRD pattern of CQDs (Fig. 7a). However, different sharp peaks were recognized at 27°, 32°, 34°, 45°, 57°, 64°, 67°, 70°, 73°, 78°, and 80° (2Ɵ) for Zn (100), (002), (101), (102), (110), (103), (200), (112), (201), (004), and (202), respectively. The observed peaks reflected the distribution of ZnO on the surface of CQDs forming CQDs/ZnO nanocomposite (Fig. 7b).

**Fig. 8:**
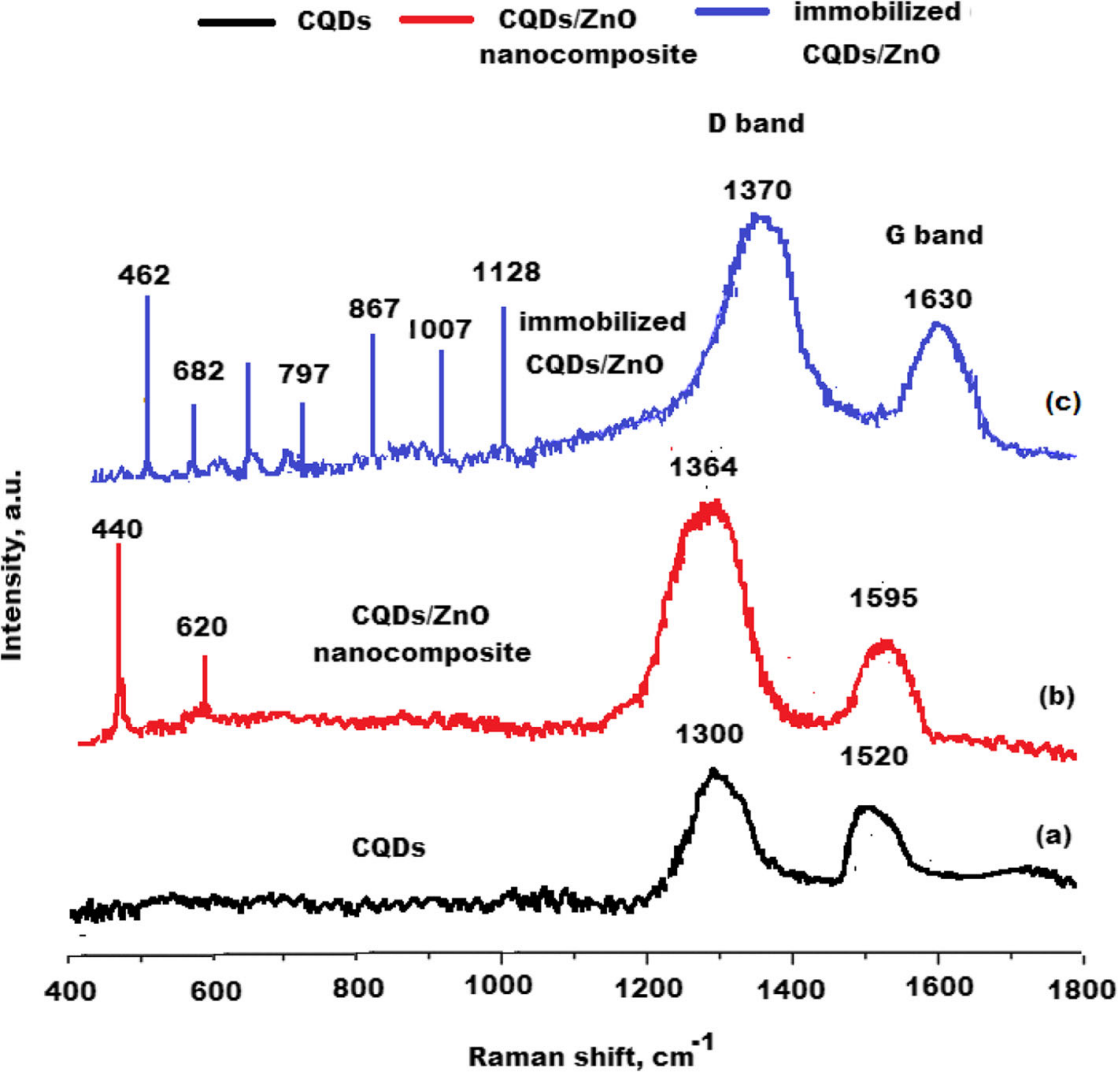
Raman spectra shift of **a** CQDs, **b** CQDs/ZnO nanocomposite, and **c** immobilized CQDs/ZnO-BM 19.21 nanocomposite

The Raman spectra of the prepared CQDs, CQDs/ZnO, and immobilized CQDs/ZnO-BM 19.21 nanocomposite were studied. The Raman signals are commonly used to study the crystal structure and its defects. Figure 8a, showed two typical D and G bands at 1300 and 1520 cm^−1^ for carbon nanoparticles, respectively. As previously reported, D-band commonly represents sp^3^ defects, and G-band is a feature of plane vibration of sp^2^-bonded carbons [44]. The *I*_*D*_/*I*_*G*_ ratio was calculated for the prepared CQDs, and it was found to be 1.02 ± 0.03. New sharp peaks were observed at 440 and 520 cm^−1^ for ZnO nanoparticles and the typical peaks of CQDs were observed at 1364 and 1595 cm^−1^. The ratio of *I*_*D*_/*I*_*G*_ was found to be 1.2 ± 0.01 indicating the formation of CQDs/ZnO nanocomposite (Fig. 8b). The Raman spectrum of immobilized CQDs/ZnO-BM 19.21 nanocomposite displayed numerous peaks that can be easily recognized as confirmatory signs of secondary and tertiary structures. The peaks observed at the region 1007–1128 cm^−1^ were assigned to represent the major secondary structure of the monoclonal antibody. The Raman peaks at 550–682 cm^−1^ region were assigned to represent disulfide conformations, whilst the 867–797 cm^−1^ ones were assigned to represent the hydrogen bonding state of tyrosine residues. Also, the significant shift in Raman spectrum peak to 1630 cm^−1^ can be attributed to the presence of tertiary structure of the immobilized antibody [45] (Fig. 8c). The ratio of *I*_*D*_/*I*_*G*_ was increased to 1.4 ± 0.04 revealing better crystalline structure due to the formation of immobilized CQDs/ZnO-BM 19.21 nanocomposite.

### Optimization of Fluorescence Immunosensing Conditions

**Fig. 9:**
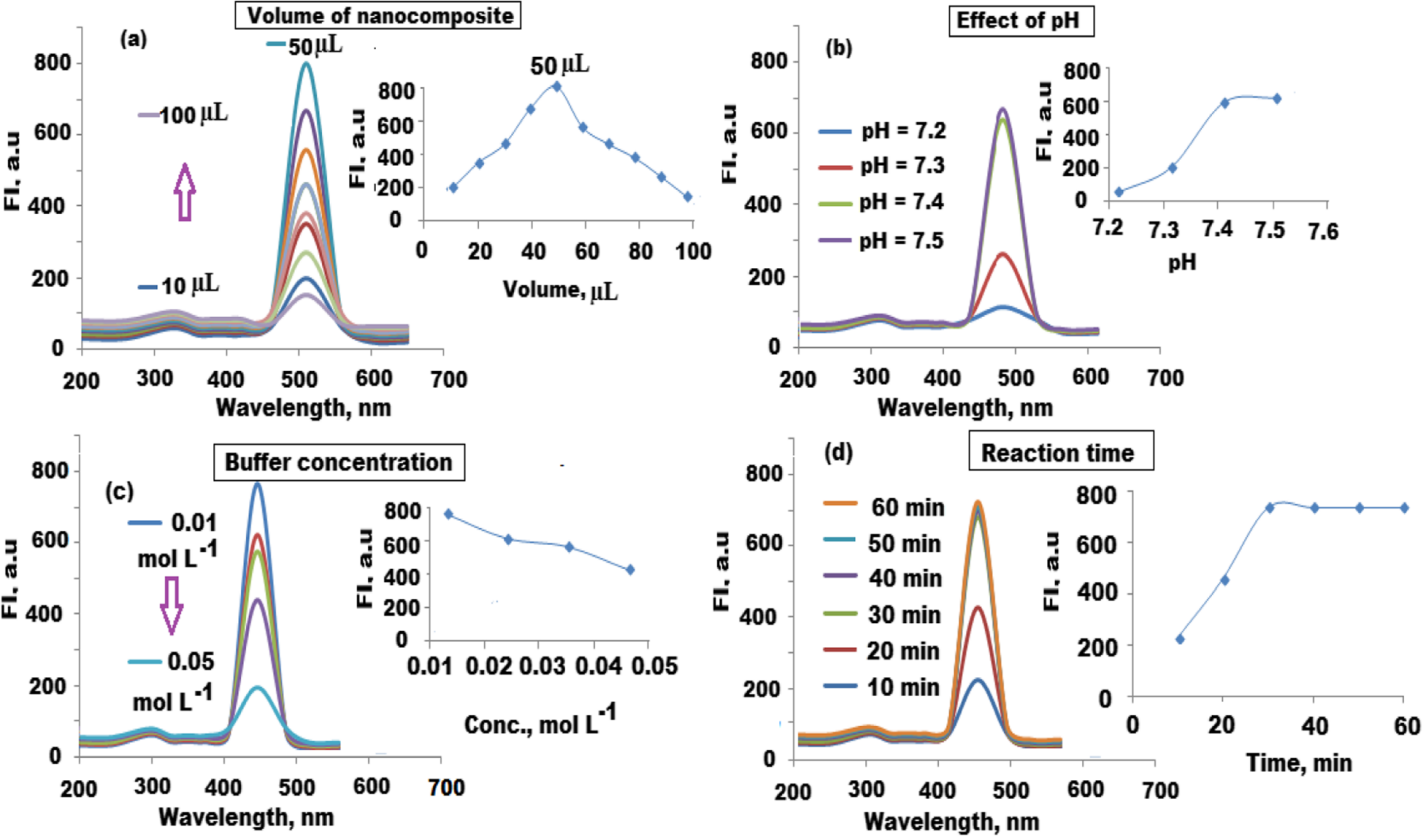
Optimization of fluorescence determination of CYFRA 21-1 antigen at λ_ex_ = 470 and λ_em_ = 520 nm. **a** Effect of added immobilized CQDs/ZnO-BM 19.21 nanocomposite, **b** effect of phosphate buffer saline of pH range 7.3–7.5, **c** effect of buffer concentration using PBS in the concentration range of 0.01–0.05 mol L^−1^, and **d** effect of immunoreaction time using 10–60 min

The selection and optimization of the suggested fluorescence immunosensing conditions were conducted by studying various parameters. Generally, the amount of immobilized nanocomposite, pH and concentration of the used buffer, incubation time between the target analyte in the serum samples, and the immunosensing reagents should be investigated and optimized. In order to select the suitable quantity of immobilized CQDs/ZnO-BM 19.21 nanocomposite, different amounts in the range of 10–100 μL were tested. The maximum fluorescence intensity was observed by adding 50 μL of the immobilized CQDs/ZnO-BM 19.21 nanocomposite (Fig. 9a). Four phosphate-buffered saline solutions of pH 7.2–7.5 values were prepared and tested as a function of fluorescence intensity. A slight change in fluorescence signal intensity was observed by changing pH values. At pH 7.2 and 7.3, the fluorescence signal was decreased due to the chemical instability of the immobilized CQDs/ZnO nanocomposite. The fluorescence signal was increased at pH 7.4 to 7.5 due to excellent interaction between the monoclonal molecules on the surface of the nanocomposite (Fig. 9b). It was found that 7.4 is the most suitable pH value to maintain the activity of target antigen which may be decomposed by increasing the pH more than 7.5. Therefore, pH 7.4 was selected for further studies.

The influence of phosphate-buffered saline concentration on the fluorescence intensity was estimated using a concentration range of 0.01–0.05 mol L^−1^. The maximum fluorescence intensity signal was obtained using the buffer concentration of 0.01 mol L^−1^. At higher buffer concentrations, the immobilized CQDs/ZnO-BM 19.21 nanocomposite was aggregated, and the instability of the immunosensing solution may cause a decrease in fluorescence intensity (Fig. 9c). To calculate the immunoreaction time, the analytical procedure was repeated using reaction time ranging from 10 to 60 min. The maximum fluorescence intensity signal was observed by maintaining the reaction between the tested antigen and the immunosensing solution for at least 30 min (Fig. 9d).

### Analytical Quantification

Under optimized conditions, the suggested immunoassay method was performed using 12 serum samples containing CYFRA 21-1 antigen in concentration range of 0.01–500 ng mL^1^. The outcome results were plotted to construct the calibration graph which was linear over a concentration range of 0.01–100 ng mL^−1^ with a detection limit of 0.008 ng mL^−1^. The calculated equation was found to be *I*_*F*_ = 7.933C + 181.24 (*r*^2^ = 0.9992). After six repetitions, the percentage of the relative standard deviation (%RSD) was 1.3%. The acceptable results revealed a high sensitivity of the immunosensing fluorescence method for the quantification of CYFRA 21-1 antigen in serum samples.

### System Suitability

System suitability was investigated by carrying out a comparative study between the suggested immobilized CQDs/ZnO-BM 19.21 immunosensing method and the previously addressed methods. The suggested fluorescence system provided significant advantages such as simplicity, eco-friendly, and easy to detect the target analyte in serum samples. The recorded results revealed high sensitivity with a wide linear detection range of 0.01–100 ng mL^1^ and lower detection limit of 0.008 ng mL^1^ (Table 1).

**Table 1:** A comparative study between the suggested immobilized CQDs/ZnO-BM 19.21 nanocomposite fluorescence immunosensing technique and previously published techniques

Technique	Principle	Linear concentration range, ng mL^−1^	Lower limit of detection, ng mL^−1^	References
Enzyme immunoassay	Enzyme immunoassay method using Boehringer Mannheim (Enzymun-Test CYFRA 21-1) in the serum	3.2–11.5	3.5	[5]
Electro-chemiluminescence immunoassay	The ability of an electrochemically luminescent molecule, a tris(2,2′-bipyridyl) ruthenium (II) complex, to be repeatedly excited by tripropylamine	3.1–10.7	3.0	[6]
Suggested immobilized CQDs/ZnO-BM 19.21 nanocomposite immunosensing system	A sandwich capping antibody-antigen-antibody fluorescence immunosensing system of immobilized CQDs/ZnO-BM 19.21	0.01–100	0.008	Present study

### Accuracy, Precision, and Selectivity of the Immobilized Immunosensing System

To ensure the accuracy of the suggested immobilized CQDs/ZnO-BM 19.21 fluorescence immunosensing system for the determination of CYFRA 21-1 antigen in serum samples, 12 serum samples were tested. The outcome data were compared with another previously reported technique [6], which was based on electrochemiluminescence assay using tris 2,2′-bipyridyl ruthenium (II) complex to be excited by tripropylamine. Acceptable results were obtained as indicated in Table 2. Intra-day and inter-day assay were used to investigate the precision of the suggested method. The test was carried out using a serum sample containing 10 ng mL^− 1^ of CYFRA 21-1 antigen. The mean relative standard deviations were 1.1% and 1.3% for both intra- and inter-day assay, respectively, which revealed high precision. Furthermore, the selectivity of the suggested method towards the determination of CYFRA 21-1 antigen was evaluated using some possible interfering species such as amino acids (cysteine, lysine, serine, tyrosine, and glycine), some cations (K^+^, Na^+^, Ca^2+^, Mg^2+^, and Zn^2+^) and some other bio-markers such as CA 15-3, CA 27-29, CA 19-9, and CA 125. The test was carried out under optimum conditions using human serum containing 10 ng mL^−1^ CYFRA 21-1 antigen in the presence of 10 ng mL^−1^ coexisting species. The outcome data were calculated as relative percentage error (Er%) and the corresponding result did not exceed ± 5% for each interfering species (Table 3). The calculated tolerance values (F-F^0^/F^0^) were found to be with the tolerance limits (< 5%). Therefore, the suggested immobilized CQDs/ZnO-BM 19.21 immunosensing fluorescence system displayed high selectivity towards the determination of CYFRA 21-1 antigen in human serum.

**Table 2:** Comparative study using immobilized CQDs/ZnO-BM 19.21 fluorescence immunosensing system and electro-chemiluminescence immunoassay [6]

Samples no.	Immobilized CQDs/ZnO-BM 19.21 fluorescence immunosensing system		Electro-chemiluminescence immunoassay [6]	
	Found, ng mL^1^	%RSD (*n* = 6)	Found, ng mL^1^	%RSD (*n* = 6)
1	0.2	± 1.3	0.1	± 1.1
2	0.6	± 0.8	0.4	± 0.5
3	1.1	± 0.5	0.9	± 1.0
4	2.0	± 0.2	1.7	± 0.6
5	2.1	± 0.4	2.0	± 0.6
6	1.8	± 0.7	1.5	± 0.2
7	0.9	± 1.0	0.6	± 1.1
8	1.4	± 0.3	1.1	± 0.7
9	2.3	± 0.1	2.1	± 0.3
10	1.5	± 1.2	1.3	± 1.4
11	1.0	± 1.3	0.7	± 1.6
12	0.7	± 1.2	0.6	± 0.7

**Table 3:** Selectivity of the suggested immobilized CQDs/ZnO-BM 19.21 method towards the determination of CYFRA 21-1 antigen in human serum

Immobilized CQDs/ZnO-BM 19.21 fluorescence method	
Interfering species, 10 ng mL^−1^	%Er
Cysteine	2.9
Lysine	3.2
Serine	1.8
Tyrosine	3.8
Glycine	2.7
K^+^	1.5
Na^+^	1.2
Ca^2+^	1.9
Mg^2+^	2.1
Zn^2+^	2.3
CA 15-3	3.5
CA 27-29	3.6
CA 19-9	2.2
CA 125	3.9

### Analysis of Real Specimens

In real human specimens, the suggested immunosensing fluorescence system based on immobilized CQDs/ZnO-BM 19.21 solution was exploiting to detect and quantify the percentage (%) recoveries of the tumor marker CYFRA 21-1 antigen. As previously mentioned in the immunosensing procedure, the suggested system was used to determine the CYFRA 21-1 antigen by finding the relationship between the fluorescence intensity and the concentration of CYFRA 21-1 antigen in serum samples. Certain amounts of the target antigen (0.5, 1.0, and 2.0 ng mL^−1^) were added to the estimated samples, and the increase in signal intensities was evaluated. After six determinations, the percentage relative standard deviations (%RSD) were calculated. The outcome percentage recoveries were found to be ranged from 96.7 ± 0.7 to 100.0 ± 1.3%. The calculated %RSD was in the range of 0.2–1.4%. The tested serum samples were analyzed using a previously reported method [6] and the percentage recoveries were found to be ranged from 96.1 ± 1.6 to 100.0 ± 0.4% with %RSD 0.3–1.7%. In order to ensure the suitability of the suggested immunosensing fluorescence technique using an immobilized CQDs/ZnO-BM 19.21 solution, a comparative statistical study using Student’s *t* test and *F* test [46] was carried out between the present results and those obtained by others from previously conducted methods (Table 4). The obtained *t* test and *F* test values were found to be ranged from 0.354 to 2.181 (2.228)* and 1.16 to 4.0 (5.05)* with respect to the tabulated values of *P* = 0.05, respectively. The results revealed good agreement between the suggested method and the previously published procedures. Also, all detected quantities of CYFRA 21-1 antigen in serum samples were within the normal limit indicating no lung cancer was diagnosed in the investigated serum samples.

**Table 4:** Comparative results obtained by the suggested Immobilized CQDs/ZnO-BM 19.21 fluorescence immunosensing system and published method [6]

Initial CYFRA 21-1 conc. ng mL^−1^	Added CYFRA 21-1, ng mL^−1^	Immobilized CQDs/ZnO-BM 19.21 fluorescence immunosensing system		Electro-chemiluminescence immunoassay^6^		t-test (2.228)**	F-test (5.05)**
		Found, ng mL^−1^	% recovery, ± %RSD*	Found, ng mL^−1^	% recovery, ± %RSD	*t* test	*F* test
0.4	0.5	0.89	98.8 ± 1.4	0.88	97.8 ± 1.6	1.157	1.31
	1.0	1.35	98.4 ± 1.2	1.36	97.1 ± 1.4	1.664	4.00
	2.0	2.38	99.2 ± 0.7	2.36	98.3 ± 0.9	1.800	1.65
1.2	0.5	1.69	99.4 ± 1.2	1.67	98.2 ± 1.5	1.534	1.56
	1.0	2.19	99.5 ± 0.7	2.20	100.0 ± 0.4	1.509	3.06
	2.0	3.13	97.8 ± 1.2	3.14	98.1 ± 1.1	0.456	1.19
2.3	0.5	2.75	98.2 ± 0.8	2.73	97.5 ± 0.6	1.716	1.78
	1.0	3.27	99.1 ± 1.1	3.25	98.5 ± 0.9	1.044	1.49
	2.0	4.29	99.7 ± 0.2	4.28	99.5 ± 0.3	1.387	2.25
0.8	0.5	1.29	99.2 ± 0.6	1.28	98.5 ± 0.9	1.618	2.00
	1.02.0	1.762.79	97.8 ± 1.499.6 ± 0.7	1.732.75	96.1 ± 1.698.2 ± 1.5	1.9662.086	1.314.59
2.0	0.5	2.46	98.4 ± 0.6	2.48	99.2 ± 0.8	2.000	1.78
	1.0	2.99	99.7 ± 0.3	2.98	99.3 ± 0.5	1.715	2.78
	2.0	3.98	99.5 ± 0.7	3.97	99.2 ± 0.4	0.905	3.06
2.1	0.5	2.58	99.2 ± 0.5	2.57	98.8 ± 0.8	1.037	2.56
	1.0	3.09	99.7 ± 0.6	3.07	99.0 ± 0.7	1.898	1.36
	2.0	4.00	97.6 ± 1.2	3.99	97.3 ± 1.7	0.354	2.01
0.7	0.5	1.18	98.3 ± 0.5	1.17	97.5 ± 0.8	2.073	2.56
	1.0	1.67	98.2 ± 0.8	1.68	99.4 ± 1.2	2.060	2.25
	2.0	2.65	98.1 ± 1.1	2.66	98.5 ± 0.9	0.687	1.49
0.5	0.5	1.00	100.0 ± 0.7	0.99	99.0 ± 0.9	2.127	1.65
	1.0	1.45	96.7 ± 1.3	1.47	98.0 ± 1.4	1.670	1.16
	2.0	2.49	99.6 ± 0.4	2.47	98.8 ± 0.8	2.181	4.00

*Number of determinations = 6

**Tabulated values of *t* test and *F* test at confidence level *p* = 0.05

## Conclusion

The present study concerned with the preparation of green synthesis CQDs conjugated with ZnO nanocomposite using *Citrus lemon* as a precursor. The CQDs/ZnO nanocomposite was employed to form a new fluorescence immunosensing system by immobilizing a monoclonal BM 19.21 antibody through simple peptide bonds. The highly sensitive fluorescence system was used to determine the tumor marker of lung cancer (CYFRA 21-1) in human serum. CYFRA 21-1 antigen was determined via sandwich capping antibody-antigen-antibody reaction using another monoclonal antibody KS 19.1 coating the microtiter wells. The unique features and high sensitivity of the suggested system facilitate the determination of the target tumor marker with high stability and reproducibility. A comparative study was carried out and the outcome results confirmed the suitability and high sensitivity of the suggested immunosensing system, and the results were in agreement with a previously reported conventional technique.

## Data Availability

The only outcome data from this study was presented in the manuscript.

## Abbreviations

%RSD: Percentage relative standard deviation
BM 19–21: Specific monoclonal antibody
CQDs: Carbon quantum dots
CQDs/ZnO: Carbon quantum dots/zinc oxide
CYFRA-21-1: Cytikeratin-19 fragment
DLS: Dynamic light scattering
EDC: Carbodiimide hydrochloride
eV: Electron volt
FT-IR: Fourier transform infrared
HRTEM: High-resolution transmission electron microscope
KS 19-1: Monoclonal cytokeratin 19-specific *antibody*
Ltd. Co: Limited company
mAb: Monoclonal antibody
NHS: N-hydroxysuccinimide
P: Degree of confidence
PBS: Phosphate-buffered saline
SEM: Scanning electron microscope
TEM: Transmission electron microscope
UK: Unite Kingdom
USA: United States of America
UV-Vis: Ultraviolet-visible
XPS: X-ray photoelectron spectroscopy
XRD: X-ray powder diffraction
ZnO: Zinc oxide
ϴ: Theta degree
λ_max_: Wavelength
